# Insights in diabetes: Molecular mechanisms-Protectin DX, an anti-inflammatory and a stimulator of inflammation resolution metabolite of docosahexaenoic acid, protects against the development of streptozotocin-induced type 1 and type 2 diabetes mellitus in male Swiss albino mice

**DOI:** 10.3389/fendo.2022.1053879

**Published:** 2023-01-25

**Authors:** Poorani Rengachar, Sailaja Polavarapu, Undurti N. Das

**Affiliations:** ^1^ BioScience Research Centre, Gayatri Vidya Parishad Institute of Healthcare and Medical Technology, Visakhapatnam, India; ^2^ Department of Microbiology, Gayatri Vidya Parishad Institute of Healthcare and Medical Technology, Visakhapatnam, India; ^3^ R&D, UND Life Sciences, Battle Ground, WA, United States; ^4^ Department of Biotechnology, Indian Institute of Technology-Hyderabad, Sangareddy, Telangana, India

**Keywords:** protectin DX, diabetes mellitus, streptozotocin, inflammation, interleukin-6, glucose, insulin

## Abstract

Our previous studies revealed that certain endogenous low molecular weight lipids have potent anti-diabetic actions. Of all, arachidonic acid (AA) and its anti-inflammatory and inflammation resolving metabolite lipoxin A4 (LXA4) are the most potent anti-diabetic molecules. Similar anti-diabetic action is also shown by resolvins. In our efforts to identify other similar lipid based anti-diabetic molecules, we investigated potential anti-diabetic action of protectin DX that also has anti-inflammatory and inducer of inflammation resolution action(s) like LXA4. Protectin DX {10(S),17(S)-dihydroxy-4Z,7Z,11E,13Z,15E,19Z-docosahexaenoic acid, also called as 10(S),17(S)-DiHDoHE)} prevented the development of streptozotocin-induced type 1 and type 2 diabetes mellitus in Swiss male albino mice. Protectin DX showed potent anti-inflammatory, antioxidant and anti-apoptotic actions that could explain its anti-diabetic action. In view of these beneficial actions, efforts need to be developed to exploit PDX and other similar compounds as potential anti-diabetic molecule in humans.

## Introduction

Diabetes mellitus (DM) is common across the globe. Of the two major types of DM, type 1 and type 2, the latter is more common. Both type 1 and type 2 DM are associated with inappropriate inflammation, immune dysfunction and gut dysbiosis.

Type 1 DM is an autoimmune disease in which pancreatic β cells are infiltrated by T cells, macrophages and NK cells that secrete excess pro-inflammatory cytokines IL-6, TNF-α, MIF (macrophage migration inhibitory factor), and IFN-γ which induce the production of excess of reactive oxygen species (ROS). Both cytokines and ROS induce apoptosis of β cells and consequently lead to the development of insulin deficient type 1 DM (1, 2). It is still uncertain as to the underlying cause for the development of type 1 DM, though undetected viral infection is one possibility (3), especially an enterovirus (for example, coxsackievirus). This assumption is supported by the reports that the number of type 1 DM patients is increased during COVID-19 pandemic (4) that have been attributed to the potential cytotoxic action of SARS-CoV-2 on pancreatic β cells, though this has been disputed. But what is certain is the worsening of hyperglycemia and increased incidence of diabetic ketoacidosis (5–7) that can be attributed to enhanced production of IL-6 and TNF-α in response to SARS-CoV-2 infection (8, 9). This is further supported by the observation that canonical cell entry factors needed for SARS-CoV-2, angiotensin-converting enzyme 2 (ACE2) and transmembrane serine protease 2 (TMPRSS2) are not co-expressed by β cells (10). In contrast to this, an increase in peripheral insulin resistance results in hyperglycemia in type 2 DM. Consumption of calorie dense diet and high fat diet (HFD) produces elevation in the production of IL-6 and TNF-α that, in turn, aggravates hyperglycemia (11–15). In addition to IL-6, TNF-α, excess production of IL-17 has also been described in those with type 1 and type 2 DM (16–19). These pro-inflammatory cytokines produced by circulating and pancreatic β cell and other tissues (including adipose tissue) infiltrating macrophages, NK cells and T cells augment generation of reactive oxygen species (ROS) produce an increase in peripheral insulin resistance in type 2 DM and induce apoptosis of β cells to lead to the development of type 1 DM (20–22). Thus, both type 1 and type 2 DM are inflammatory conditions implying that methods designed to ameliorate inflammation may form a new therapeutic approach to DM.

Previously, we showed that low molecular weight lipid molecules such as linoleic acid (LA), alpha-linolenic acid (ALA), gamma-linolenic acid (GLA), dihomo-GLA (DGLA), arachidonic acid (AA), eicosapentaenoic acid (EPA) and docosahexaenoic acid (DHA) have potent anti-diabetic actions especially against chemical (alloxan and streptozotocin)-induced type 1 DM (23–26). Of all the fatty acids tested, AA is the most potent (AA > DHA > DHA > GLA > DGLA > LA > ALA) in preventing chemical-induced DM (especially type 1 DM). In an extension of this study, we observed that the anti-diabetic action of AA is due to its potent anti-inflammatory action as evident from its ability to suppress IL-6 and TNF-α production and the expression of NF-κB (27, 28). Administration of AA enhanced the formation of its anti-inflammatory metabolite lipoxin A4 (LXA4) that seems to be responsible for the anti-diabetic action of AA (27–29). It is noteworthy that alloxan, STZ, doxorubicin and benzo(a)pyrene (BP) inhibited the production of both BDNF and LXA4 by pancreatic β cells *in vitro* and animals with STZ-induced type 2 DM have low plasma levels of LXA4 and BDNF (30). These results are in support of the observation that plasma levels of BDNF and LXA4 are low in those who are at high risk of developing DM and patients with type 2 DM (29, 31–33). Thus, there is a close relationship among EFAs and their metabolites, inflammation, insulin resistance, β cell apoptosis and DM. These beneficial actions of AA, LXA4 and BDNF in the prevention and management of DM could be ascribed to their anti-inflammatory and ability to induce resolution of inflammation actions (27–35). This is supported by the observation that resolvins, anti-inflammatory and inducers of resolution of inflammation metabolites formed from EPA ad DHA, can also prevent chemical-induced type 1 and type 2 DM in experimental animals (36, 37). These results emphasize the significance of endogenous anti-inflammatory and inducers of resolution of inflammation molecules as potential anti-diabetic compounds. To further evaluate this concept, in the present study, we evaluated the effect of protectin DX, an anti-inflammatory and inducer of resolution of inflammation metabolite formed from DHA in the prevention of STZ-induced type 1 and type 2 DM in Swiss male albino mice and its potential mechanism of action.

## Materials and methods

### Chemicals

All reagents and chemicals were purchased from Sigma Aldrich Chemical Pvt Ltd (USA). 10(S), 17(S) DiHDHA (protectin DX, henceforth called as PDX) was obtained from Cayman Chemical Company (USA). ELISA kits were procured from BioLegend (USA).

### Experimental animals

6-8 weeks old male Swiss albino mice procured from National Institute of Nutrition (NIN, Hyderabad, India) were used in this study. Animals were acclimatised for a week before the start of the study. The animals were housed at 25 ± 2^0^C room temperature with 12-hr dark and 12-hr light cycle. The study was approved by the Institutional Animal Ethics Committee.

### Type 1 DM study

**Figure 1:**
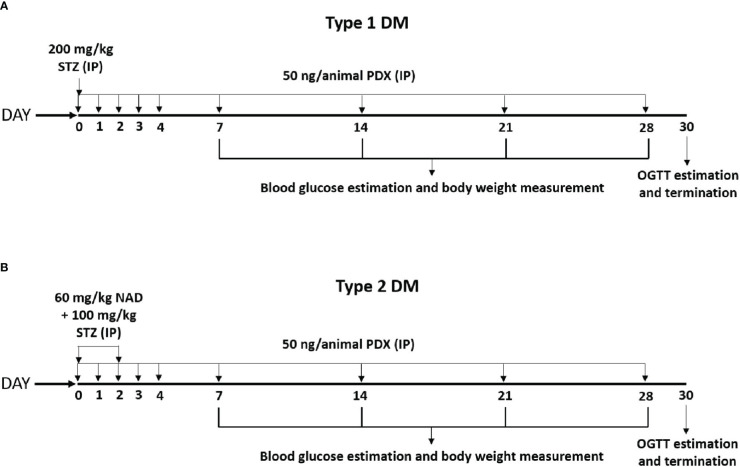
Experimental plan for STZ induced Type 1 and Type 2 diabetes mellitus (DM) in male Swiss albino mice. **(A)** Type 1 DM protocol – Diabetes was induced by a single dose of STZ (200 mg/kg body weight, IP) on day 0. These animals received PDX (50 ng/animal, i.p.) simultaneously along with STZ on day 0 and for the next 5 consecutive days (Day 0 to Day 4). The STZ + PDX group also received PDX every week for the next 4 weeks [as shown in the **(A)**]. Experiment was terminated and animals were sacrificed on Day 30 following OGTT estimation. **(B)** Type 2 DM protocol – Diabetes was induced by the administration of STZ (100 mg/kg body weight, i.p.) on day 0 and day 2. NAD (60 mg/kg body weight) was injected 15 min prior to STZ administration on day 0. STZ + PDX animals received PDX (50 ng/animal i.p.) on day 0 and the following 4 consecutive days (Day 0 to Day 4). Subsequently STZ +PDX group received PDX (50 ng/animal i.p.) once every week for the next 4 weeks. Experiment was terminated and animals were sacrificed on Day 30 following OGTT estimation [see **(B)**]. STZ, Streptozotocin; PDX, 10(S),17(S)-DiHDoHE; NAD, Nicotinamide; OGTT, Oral Glucose Tolerance Test; i.p., intraperitoneal.

Animals weighing ~30 g were divided into 4 groups with 6 animals in each group: control, diabetic (Type 1DM group), PDX and PDX + Type 1DM. Control group received PBS as vehicle. T1DM was induced by a single dose of streptozotocin (STZ, 200 mg/kg body weight given intraperitoneally) on day 0. PDX group was treated initially with PDX (50 ng/animal, i.p.) for 5 consecutive days (Day 0 to Day 4) and subsequently once in a week for the next 4 weeks. PDX + Type 1DM group received STZ and PDX simultaneously on day 0 and subsequently once in a week for the next 4 weeks (see **Figure 1A**). Body weight, food intake and water intake were noted on alternate days. Fasting blood glucose was measured using Accu-Check blood glucose meter (Roche, USA) on 7^th^, 14^th^, 21^st^ and 28^th^ day of the study. Experiment was terminated on 30^th^ day of the study after OGTT estimation. Blood was collected by cardiac puncture. Plasma and pancreas organ obtained and stored at -80°C for further analysis.

### Type 2 diabetes studies

Animals weighing ~30 g were separated into 4 groups with each group containing 6 animals: control, diabetic (Type 2DM), PDX and PDX + Type 2DM. Control group received PBS as vehicle. Type 2 DM was induced by administration of STZ (100 mg/kg body weight, i.p.) on day 0 and day 2. Nicotinamide (NAD, 60 mg/kg body weight) was injected 15 min prior to STZ administration on day 0. PDX group received PDX (50 ng/animal, IP) for 5 consecutive days (Day 0 to Day 4) and subsequently once in week for 4 weeks. PDX + Type 2DM group received simultaneously NAD, STZ and PDX (**Figure 1B**). Control animals received only the vehicle. Body weight, food intake and water intake were noted every alternate day. Fasting blood glucose was measured using Accu-Check blood glucose meter (Roche, USA) on 7^th^, 14^th^ 21^st^ and 28^th^ day. Experiment was terminated and animals were sacrificed on 30^th^ day following OGTT estimation. Blood was collected by cardiac puncture. Separated plasma and pancreas were stored at -80°C for further analysis.

### Estimation of Oral Glucose Tolerance Test (OGTT) and Insulin Sensitivity Indices (ISI)

At the end of study, OGTT was performed in all group of animals following overnight fasting in both Type 1 DM and Type 2 DM experiments. After the collection of the fasting blood sample (considered time point 0), the animals were given an oral glucose load (2 g/kg) by gavage. Further blood samples were collected at the end of 30, 60, 90 and 120 min from the retro orbital plexus. ISI was computed by HOMA IR, HOMA B and QUICKI (Quantitative insulin check index) methods in Type 1 DM and Type 2 DM studies.

### Analysis of antioxidant enzymes, lipid peroxides and nitric oxide

Plasma and pancreatic lysates were used for the estimation of antioxidant enzymes: catalase, superoxide dismutase (SOD), glutathione-S-transferase (GST), glutathione peroxidase (GPX) as described previously (23–28). Lipid peroxides (LPO) was estimated as malondialdehyde (MDA) formed on reaction with thiobarbituric acid (TBA) and nitric oxide (NO) measured as nitrite formed using Griess reagent. Both LPO and NO estimated in the plasma as described previously (23–28).

### Plasma analysis for various cytokines by ELISA

Mice plasma samples were analysed for the concentrations of insulin (Mouse Insulin ELISA, ALPCO, USA, 80-INSMS-E01, E10), TNF-α (Mouse TNF-α ELISA, BioLegend, USA, 430901), IL-6 (Mouse IL-6 ELISA, BioLegend, USA, 431301) and IL-10 (Mouse IL-10 ELISA, BioLegend, USA, 431411) as per manufacturer instructions.

### Gene expression studies

Total RNA was isolated from mice pancreatic tissue by using Trizol method; cDNAs were synthesized by reverse transcriptase from 1 µg of total RNA using SuperScript First-Strand Synthesis for qRT–PCR (Invitrogen) kit by following manufacturer instructions. Semiquantitative Polymerase Chain Reaction (PCR) was performed to study the expression of Bcl2, Bax, NFκB, IκB, iNOS and β-actin as follows: 94°C for 2 min initial denaturation, 94°C for 30 s denaturation, 30 s annealing, followed by 72°C for 30 s extension and 72°C for 5 min final extension, and overall, 35 cycles were performed. PCR products were observed and analysed by electrophoresis on 1.5% (w/v) agarose gel in 1X TAE buffer at 100V. Quantification was performed by taking the ratio of gene of interest and β-actin and calculated as percentage comparing with the respective control. The details of primer genes, respective annealing temperatures and product size are mentioned in **Table 1**. PCR was performed in BlueRay Biotech TurboCycler. Quantification of genes was done by Major Science image analysis software.

**Table 1:** Details of primer genes, product size and their respective annealing temperatures used in semiquantitative polymerase chain reaction.

Gene	Primer Pair (5' - 3')	Product Size (bp)	Annealing Temperature
β-actin	S: CGTGGGCCGCCCTAGGCACCA A: TTGGCCTTAGGGTTCAGGGGGG	243	52.5°C
Bax	S: CGGCGAATTGGAGATGAACTG A: GCAAAGTAGAAGAGGGCAACC	161	58°C
Bcl-2	S: CTCGTCGCTACCGTCGTGACTTCG A: CAGATGCCGGTTCAGGTACTCAGTC	242	66°C
NF-κB	S: CAGCACTGATGGCATGGGGGACACTGACA A:CCCAATGCATAGCCATTACACGTTTTTCACCTTAAATCTGCTT	588	68°C
IκB	S: CTTGGTGACTTTGGGTGCTGAT A: GCGAAACCAGGTCAGGATTC	101	59°C
iNOS	S: CCCTTCCGAAGTTTCTGGCAGCAGC A: GGCTGTCAGAGAGCCTCGTGGCTTTGG	499	60°C

S, Sence sequence; A, antisense sequence; bp. base pairs.

### Statistical analysis

Experiments were performed by taking six animals in each group. Analysis was done twice in triplicate to ensure the repeatability of the results obtained. All results are expressed as mean ± SEM. The obtained values were analysed by paired t-test with equal variance using Microsoft Excel statistical analysis tool. Additionally, the results were also analysed using ANOVA test wherever necessary which showed that the statistical significance was like the Student’s T test.

## Results

### Effect of PDX on body weight in Type 1 and Type 2 DM animals

**Figure 2:**
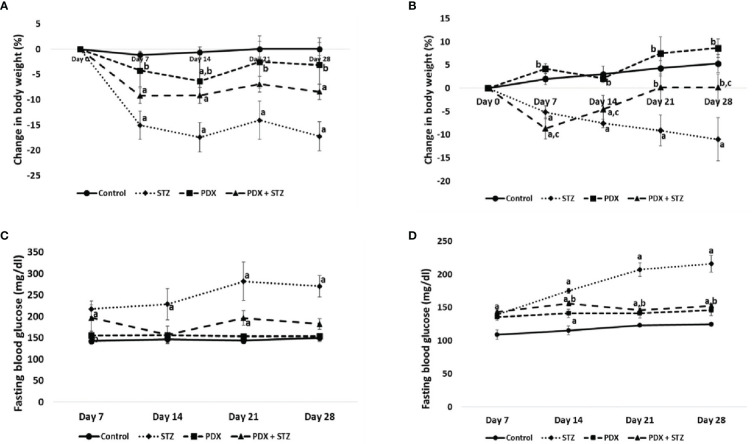
Change in body weight expressed in percentage (%) in Type 1 DM **(A)** and Type 2 DM **(B)**. Fasting blood glucose levels in Type 1 DM **(C)** and Type 2 DM **(D)**. All values are expressed in mean + SEM. N=6. a,b,c p < 0.05 compared to control, STZ and PDX respectively. STZ, Streptozotocin; PDX, 10(S),17(S)-DiHDoHE.

STZ-induced type 1 and type 2 DM animals showed significant 17% and 11% reduction in bodyweight respectively by 28^th^ day of the study. In contrast, treatment with PDX improvement bodyweight by 9% in Type 1 DM animals (**Figure 2A**) on 28^th^ day of the study and complete restoration of weight to near normal by 21^st^ day in Type 2 DM animals (**Figure 2B**).

### Effect of PDX treatment on blood glucose in Type 1 and Type 2 DM animals

The fasting blood glucose concentrations were consistently elevated in both STZ-induced type 1 and type 2 DM animals throughout the study. At the end of the study, fasting blood glucose levels were around 270mg/dl in Type 1 DM and 240mg/dl in Type 2 DM animals. It is noteworthy that PDX treatment restored (p<0.05) fasting blood glucose levels to near normal levels in both Type 1 (182 mg/dl, **Figure 2C**) and Type 2 (167 mg/dl, **Figure 2D**) DM animals. PDX treatment alone did induce any significant change in the plasma glucose levels. In addition, we did not observe any noticeable side effects in the PDX treated animals (both PDX control and STZ + PDX groups) (see **Figures 2C, D**).

### Effect of PDX treatment on OGTT

PDX treatment produced significant reduction (p<0.05) in both glucose intolerance and insulin resistance tests (**Table 2**) in both Type 1 and Type 2 DM animals. The OGTT studies showed that PDX treatment can induce a significant reduction in the plasma blood glucose levels in Type 1 DM animals and restored to normal the plasma blood glucose levels in Type 2 DM animals (**Table 3**). These results imply that PDX treatment can reduce insulin resistance.

### Effect of PDX on antioxidants, lipid peroxides and nitric oxide concentrations in STZ-induced Type 1 and Type 2 DM animals

The results of this study showed in **Table 4** revealed that that PDX treatment can restore catalase and GST, NO and LPO levels in the plasma and pancreas tissue to near normal levels in both Type 1 and Type 2 DM animals except for superoxide dismutase (SOD) levels that was restored near to normal only in the pancreatic tissue (**Table 4**). It is noteworthy that PDX alone led to a significant a decrease in pancreatic NO and an increase in pancreatic SOD and GST levels compared to control (see **Table 4**) in type 1 DM animals. But otherwise, PDX treatment led to restoration of all the STZ-induced alterations in plasma and pancreatic NO, LPO, catalase, SOD, GST and GPX values to near normal in both type 1 and type 2 DM animals (see **Table 4**). It is interesting that PDX treatment enhanced pancreatic SOD activity significantly higher than control (control: 3.13 ± 0.51 *vs* STZ: 14.10 ± 2.07 *vs* PDX + STZ: 6.11 ± 0.89) in type 1 DM animals. In a similar fashion, PDX treatment reduced significantly levels of plasma NO in PDX + STZ group (control: 20.65 ± 1.43 *vs* PDX + STZ: 15.22 ± 1.92); plasma LPO (control: 1.13 ± 0.01 *vs* 0.81 ± 0.12) and enhanced plasma SOD (control: 2.66 ± 0.25 vs PDX + STZ: 5.42 ± 0.10) and GST (control: 1.47 ± 0.11 *vs* PDX + STZ: 2.22 ± 0.22) activities and reduced pancreatic LPO levels (control: 3.79 ± 0.10 *vs* PDX + STZ: 3.04 ± 0.29); and reduced SOD (control 23.12 ± 1.51 *vs* PDX + STZ: 15.79 ± 1.68); GST (control: 6.10 ± 0.22 *vs* PDX + STZ: 4.58 ± 0.44) and GPX activities (control: 5122.24 ± 337.69 *vs* PDX + STZ: 3822.27 ± 232.91) significantly in type 2 DM animals (see **Table 4**). These results suggest that PDX treatment can reset pro-oxidant and antioxidant status (p<0.05) and thus, counters the oxidative stress induced by STZ treatment.

### Effect of PDX treatment on the concentrations of insulin and cytokines in STZ-induced Type 1 and Type 2 DM animals

**Figure 3:**
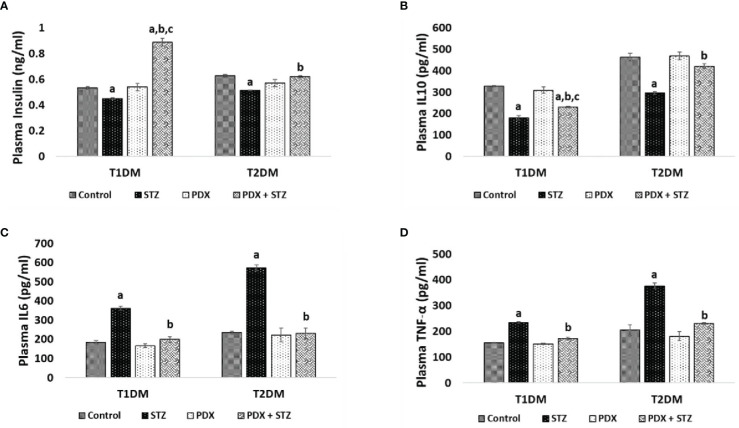
ELISA estimations in mice plasma. Insulin **(A)**, IL10 **(B)**, IL6 **(C)**, TNF-α **(D)** were estimated in Type 1 DM and Type 2 DM mice plasma using ELISA. All values are expressed in mean + SEM. N=6. a,b,c p < 0.05 compared to control, STZ and PDX respectively. STZ – Streptozotocin, PDX – 10(S),17(S)-DiHDoHE, T1DM – Type 1 diabetes mellitus, T2DM – Type 2 diabetes mellitus.

STZ treatment produced a significant decrease in plasma insulin and IL-10 levels and a significant increase in IL-6 and TNF-α levels which suggests an overall increase in inflammatory status in both STZ-induced Type 1 and Type 2 DM mice. Treatment with PDX significantly increased plasma insulin and IL-10 levels while inducing a decrease in IL-6 and TNF-α levels in both STZ-induced Type 1 and Type 2 DM mice (**Figure 3**). It is interesting that plasma insulin levels were significantly increased compared to control in type 1 DM animals. In contrast, type 2 DM animals showed restoration of plasma insulin levels to control level (see **Figure 3A**). It is observed that plasma IL-10, IL-6 and TNF-α levels were restored to near normal levels in both type 1 DM and type 2 DM animals

(see **Figures 3B–D**). The dramatic increase in plasma insulin levels in type 1 DM animals treated with PDX (PDX + STZ) is rather interesting. It is noteworthy that the increase in plasma IL-6 and TNF-α were much higher in the STZ-treated animals in the type 2DM group compared to the increases seen in type 1 DM group that were also restored to normal following PDX treatment (see **Figures 3C, D**). These results suggest that PDX has potent anti-inflammatory actions.

### Effect of PDX on the expression of Bax, Bcl-2, NFkB, iNOS genes

**Figure 4:**
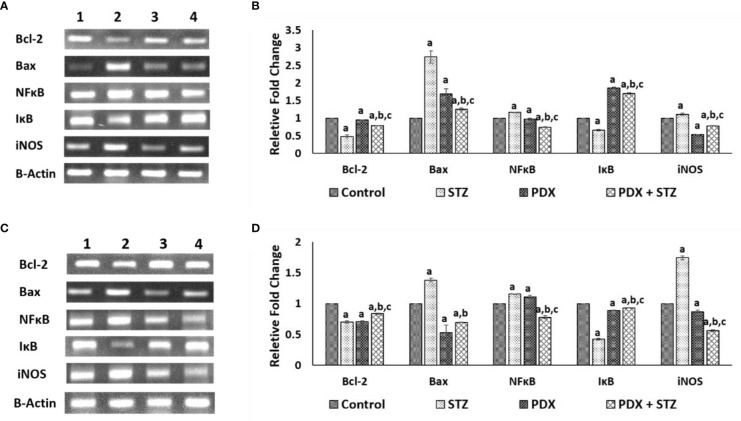
Gene expression studies in Type 1 DM and Type 2 DM mice pancreatic tissue. Expression of anti-apoptotic gene: Bcl-2, apoptotic gene: Bax in Type1 DM **(A, B)** and Type 2 DM **(C, D)** mice pancreatic tissue when treated with PDX and STZ simultaneously. The levels of all genes quantified by densitometric analysis. Gene expressions were normalized by β-actin gene expression. (1) Control, (2) STZ, (3) PDX, (4) PDX + STZ. All values are expressed in mean + SEM. N=3. a,b,c p < 0.05 compared to control, STZ and PDX respectively. STZ, Streptozotocin; PDX, 10(S),17(S)-DiHDoHE.

STZ treatment caused a significant decrease in Bcl-2 with a concomitant increase in Bax expression in pancreatic tissue that was restored to near normal by PDX treatment in both STZ-induced Type 1 (**Figures 4A, B**) and Type 2 (**Figures 4C, D**) DM animals. Similar trend was observed with respect to pro-inflammatory gene NFκB (increasing significantly) and anti-inflammatory gene IκB expression (decreased) upon treatment with STZ which was reverted to near normal by PDX treatment in both STZ-induced Type 1 (**Figures 4A, B**) and Type 2 (**Figures 4C, D**) DM animals. STZ treatment induced a significant increase in the expression of iNOS which was reduced by PDX treatment in both STZ-induced Type 1 (**Figures 4A, B**) and Type 2 (**Figures 4C, D**) DM animals. These results are in tune with the plasma and pancreatic NO levels observed in the present study (see **Table 4**).

## Discussion

In view of the increasing incidence of obesity and DM, it is imperative to develop effective methods of preventing and managing these diseases. Both type 1 and type 2 DM are inflammatory conditions (1, 2, 16–22) implying that anti-inflammatory and β cell protective compounds could be of benefit in preventing DM. Our previous studies revealed that AA, LXA4, resolvins and protectins prevent β cell cytotoxic action of alloxan and STZ *in vitro* and prevented or reduced the severity of both type 1 and type 2 DM in experimental animals (23–29), which can be attributed to their anti-inflammatory and anti-apoptotic (or cytoprotective) actions (27–30, 37–39).

The results of the present study showed that protectin DX can prevent development of both type 1 and type 2 DM in addition to restoring plasma insulin levels, food and water intake and bodyweight (see **Figure 2** and **Table 5**); plasma levels of IL-6, IL-10, TNF-α (see **Figure 3**); expression of Bcl-2/Bax, iNOS, NF-kB and IkB genes (see **Figure 4**); balance between pro- and anti-oxidants (**Table 4**); OGTT (**Table 3**) and insulin indices (**Table 2**) to near normal. These results indicate that PDX can restore to near normal all the indices that are altered by STZ treatment. It is noteworthy that PDX is effective against both type 1 and type 2 DM induced by STZ treatment though all indices (especially plasma glucose, insulin, IL-6, IL-10 and TNF-α and pro- and antioxidants levels) measured were more significantly elevated (or altered) in type 1 DM group compared to type 2 DM. This is as expected since inflammation is more severe in type 1 DM compared to type 2 DM animals. The better response seen in STZ-induced type2 DM animals compared to STZ-induced type 1 DM group is expected since inflammation and pancreatic β cell damage is more in type 1 DM animals. A higher HOMA-IR value indicates greater insulin resistance (IR), and a lower HOMA-B value indicates greater β-cell dysfunction. It is evident from the data shown in **Table 2** that STZ-induced T1 and T2 DM animals have almost same degree of insulin resistance (HOMA-IR) and the response to protectin DX is also similar, while β cell dysfunction (measured as HOMA-B) is higher in T1 DM (control: 44.44 ± 0.74; STZ: 18.14 ± 0.80; PDX: 49.44 ± 1.68; STZ + PDX: 40.05 ± 4.50) compared to type 2 DM group (control: 85.98 ± 3.38; STZ: 27.54 ± 1.92; STZ + PDX: 58.55 ± 5.92; STZ + PDX: 58.68 ± 2.46). Protectin DX treatment improved β cell function in both T1 and T2 DM animals but is more significant in T1 DM group. STZ-induced T2DM animals showed significant improvement in β cell dysfunction but was much less compared to T1DM group. This data suggests that PDX is more effective in restoring β cell dysfunction in type 1 DM compared to type 2 DM. These beneficial actions of PDX against IR and β cell dysfunction could be attributed to its anti-inflammatory action and inflammation resolution function.

**Table 2:** Insulin sensitivity index (ISI) in STZ-induced Type 1 DM and Type 2 DM mice.

		HOMA IR	HOMAB	QUICKI
T1DM	Control	4.97 ± 0.10	44.44 ± 0.74	0.49 ± 0.002
	STZ	6.94 ± 0.31*^a^*	18.14 ± 0.80*^a^*	0.45 ± 0.004*^a^*
	PDX	4.73 ± 0.14	49.44 ± 1.68*^a^*	0.49 ± 0.003
	PDX ± STZ	5.37 ± 0.52*^b^*	40.05 ± 4.50*^b^*	0.48 ± 0.009*^b^*
T2DM	Control	4.46 ± 0.11	85.98 ± 3.38	0.50 ± 0.003
	STZ	6.53 ± 0.39*^a^*	27.54 ± 1.92*^a^*	0.46 ± 0.005*^a^*
	PDX	4.85 ± 0.36	58.55 ± 5.92*^a^*	0.49 ± 0.008
	PDX ± STZ	5.38 ± 0.13*^a^*,*^b^*	58.68 ± 2.46*^a^*,*^b^*	0.48 ± 0.002*^a^*,*^b^*

ISI was computed by HOMA IR, HOMA B and QUICKI (Quantitative insulin check index) methods in Type 1 DM and Type 2 DM mice. All values are expressed in mean ± SEM. N=6. **^a,b^** p < 0.05 compared to control and STZ. STZ, Streptozotocin; PDX, 10(S),17(S)-DiHDoHE; T1DM, Type 1 diabetes mellitus; T2DM, Type 2 diabetes mellitus.

**Table 3:** OGTT in STZ-induced Type 1 DM and Type 2 DM mice.

	Group	0 min	30 min	60 min	90 min	120 min
T1DM	Control	150.50 ± 8.96	289.33 ± 23.56	280.33 ± 57.72	165.33 ± 5.57	164.00 ± 10.01
	STZ	270.50 ± 28.68^a^	280.00 ± 2.31	316.50 ± 0.87	434.00 ± 64.09 ^a^	322.50 ± 33.20^a^
	PDX	154.00 ± 2.54	210.00 ± 15.37 ^b^	177.33 ± 5.92 ^b^	191.00 ± 15.98 ^b^	186.33 ± 13.19 ^b^
	PDX ± STZ	182.20 ± 11.57^b^	242.33 ± 5.27 ^b^	243.33 ± 13.42^b,c^	223.33 ± 20.40 ^b^	221.67 ± 25.59 ^b^
T2DM	Control	149.33 ± 4.73	309.00 ± 19.53	245.33 ± 16.40	177.00 ± 8.81	159.33 ± 1.53
	STZ	243.67 ± 12.33^a^	570.00 ± 17.35 ^a^	491.67 ± 36.67 ^a^	404.00 ± 17.23 ^a^	335.00 ± 5.89 ^a^
	PDX	173.67 ± 9.61 ^b^	192.00 ± 5.49 ^a,b^	217.67 ± 11.23 ^b^	198.33 ± 21.09 ^b^	188.67 ± 7.88 ^b^
	PDX ± STZ	167.33 ± 5.03 ^b^	274.33 ± 22.07 ^b^	184.00 ± 14.18 ^b^	156.00 ± 7.81 ^b^	153.00 ± 8.56 ^b^

All values are expressed in mean ± SEM. N=6. **^a,b,c^** p < 0.05 compared to control, STZ and PDX respectively. STZ, Streptozotocin; PD, 10(S),17(S)-DiHDoHE; T1DM, Type 1 diabetes mellitus; T2DM, Type 2 diabetes mellitus.

**Table 4:** Estimation of nitric oxide, lipid peroxidation and anti-oxidant status in Type 1 DM and Type 2 DM mice.

		GROUPS	NO (µM)	LPO (µM)	Catalase (µM H202/min/ gm protein)	SOD (Units/mg protein)	GST (µM/min/gm protein)	GPX(µM/min/gm protein)
T1DM	Plasma	Control	18.82 ± 0.38	2.25 ± 0.18	16.11 ± 2.40	9.51 ±2.24	0.96 ±0.09	175.55 ±11.56
		STZ	30.57 ± 0.72^a^	4.25 ± 0.07 ^a^	50.73 ± 5.10 ^a^	5.87 ± 0.36	1.44 ± 0.18 ^a^	148.31 ± 10.90
		PDX	20.65 ± 1.13 ^b^	2.03 ± 0.09 ^b^	20.64 ±3.12 ^b^	10.40 ± 2.39	0.96±0.08 ^b^	153.79 ± 7.49
		PDX ±STZ	20.71 ±0.93 ^b^	2.73 ± 0.52 ^b^	14.16 ±1.09 ^b^	10.63 ± 5.98	0.80 ± 0.04 ^b^	146.06 ± 7.67
	Pancreas	Control	14.52 ± 1.02	5.36 ± 0.06	46.49 ±4.80	3.13 ±0.51	5.08 ±0.46	486.48 ± 37.89
		STZ	27.22 ± 0.97 ^a^	6.52 ± 0.19 ^a^	131.72 ± 7.43 ^a^	14.10 ± 2.07 ^a^	5.80 ± 0.52	393.20 ± 25.45
		PDX	9.46 ± 1.69 ^a,b^	5.46 ± 0.39 ^b^	43.50 ±3.03 ^b^	11.41 ±1.51 ^a^	6.98 ±0.50 ^a^	458.66 ± 48.27
		PDX ±STZ	12.56 ± 2.29 ^b^	4.93 ± 0.27' ^b^	52.15 ±8.72 ^b^	6.11±0.89 ^a,b,c^	5.49 ± 0.94	404.67 ± 22.80
T2DM	Plasma	Control	20.65 ± 1.43	1.13 ± 0.03	8.01 ±1.29	2.66 ±0.25	1.47 ± 0.11	677.54 ±43.32
		STZ	26.34 ±0.22 ^a^	1.97 ± 0.15 ^a^	21.61 ± 4.73 ^a^	2.09 ± 0.43	2.32 ± 0.31 ^a^	683.10 ± 40.49
		PDX	20.15 ±0.69 ^b^	1.26 ± 0.06 ^b^	9.64 ±0.90 ^b^	5.27 ±1.20 ^b^	1.63 ±0.11 ^b^	789.20 ± 61.93
		PDX±STZ	15.22 ±1.92 ^a,b,c^	0.81±0.12 ^a,b,c^	9.77 ±0.09 ^b^	5.42 ±0.10 ^a,c^	2-21 ± 0.22 ^a,c^	808.18 ± 90.00
	Pancreas	Control	5.55 ± 0.71	3.79 ± 0.10	52.38 ± 1.13	23.12 ± 1.51	6.10 ± 0.27	5123.24 ± 337.69
		STZ	8.39 ± 0.86 ^a^	4.85 ± 0.13 ^a^	77.44 ± 8.10 ^a^	33.99 ±2.59 ^a^	8.54 ±0.37 ^a^	9438.47±1152.06 ^a^
		PDX	5.74 ± 0.64 ^b^	3.75 ± 0.24 ^b^	54.47 ± 5.14 ^b^	19.18 ± 5.30 ^b^	6.21 ± 0.88 ^b^	6479.07 ± 644.73 ^b^
		PDX±STZ	5.10 ± 0.91 ^b^	3.04 ± 0.29 ^a,b^	36.48 ± 7.30 ^b^	15.79 ± 1.68 ^a,b^	4.58 ± 0.44 ^a,b^	3822.27 ± 232.91 ^a,b,c^

All values are expressed in mean ± SEM. N=6. **^a,b,c^** p < 0.05 compared to control, STZ and PDX respectively. STZ, Streptozotocin; PDX, 10(S),17(S)-DiHDoHE; T1DM, Type 1 diabetes mellitus; T2DM, Type 2 diabetes mellitus.

**Table 5:** Measurement of food and water intake of Type 1 DM and Type 2 DM mice.

	Parameter	Group	Week 1	Week 2	Week 3	Week 4
T1DM	Food Intake (gm)	Control	25.29 ± 2.87	24.76 ± 2.58	26.68 ± 2.08	27.00 ± 1.75
		STZ	22.29 ± 2.03	29.30 ± 2.90	32.79 ± 2.30	32.50 ± 1.76
		PDX	25.57 ± 3.11	24.76 ± 2.54	26.89 ± 2.17	27.15 ± 1.84
		PDX ± STZ	30.86 ± 4.60	36.23 ± 3.80	43.94 ± 3.80	43.62 ± 3.00
	Water Intake (ml)	Control	42.14 ± 2.40	39.64 ± 1.82	36.67 ± 1.72	35.00 ± 1.37
		STZ	89.29 ± 11.97	84.64 ± 6.29	84.04 ± 4.32	79.14 ± 3.76
		PDX	37.14 ± 1.84	34.64 ± 1.52	33.33 ± 1.35	32.07 ± 1.15
		PDX ± STZ	60.71 ± 6.68	65.00 ± 3.88	68.09 ± 3.03	65.86 ± 2.57
T2DM	Food Intake (gm)	Control	36.00 ± 0.52	35.67 ± 4.67	30.50 ± 1.34	41.56 ± 8.08
		STZ	42.84 ± 4.05	58.05 ± 5.00	49.67 ± 1.36	52.06 ± 10.20
		PDX	34.67 ± 3.27	36.83 ± 4.20	35.33 ± 0.33	41.89 ± 8.33
		PDX ± STZ	37.00 ± 4.91	54.17 ± 4.15	52.17 ± 2.47	57.58 ± 11.03
	Water Intake (ml)	Control	32.14 ± 4.61	25.00 ± 1.54	47.86 ± 6.71	50.00 ± 1.89
		STZ	37.86 ± 5.44	32.86 ± 2.41	44.29 ± 3.35	53.57 ± 1.43
		PDX	32.86 ± 5.86	29.29 ± 1.70	35.00 ± 1.89	45.71 ± 1.70
		PDX ± STZ	37.86 ± 3.43	35.00 ± 1.89	42.14 ± 1.01	52.86 ± 1.01

All values are expressed in mean ± SEM. N=6. STZ, Streptozotocin; PDX, 10(S),17(S)-DiHDoHE; T1DM, Type 1 diabetes mellitus; T2DM, Type 2 diabetes mellitus.

Previous studies suggested that DHA, the precursor of PDX, when used at 30 μM not only protected against palmitate (500 μM)-induced cytotoxicity but also prevented insulin resistance in C2C12 myotubes by decreasing protein kinase C (PKC)-θ activation and restoring cellular acylcarnitine profile, insulin-dependent AKT phosphorylation and glucose uptake. It was reported that DHA suppressed the production of IL-6 and TNF-α that is responsible for its beneficial action. In experimental animals that were fed high-cholesterol–high-sucrose diet, supplementation with DHA enhanced the insulin-dependent AKT phosphorylation and reduced the PKC-θ activation in skeletal muscle. These results imply that DHA prevents lipotoxicity and inflammation (40). It was reported that DHA can restore pancreatic duodenal homeobox-1 (PDX1) expression levels and β-cell function induced by palmitic acid by inhibiting elevation of proinflammatory chemokines and activation of NF-kB, c-Jun amino (N)-terminal kinases1/2 and p38MAPK signalling pathways. These actions of DHA seem to be mediated by GPR120. DHA supplementation ameliorated glucose tolerance and insulin sensitivity, and improved *Pdx1* expression and islet inflammation in diet-induced obese mice indicating that GPR120 activation is protective against lipotoxicity-induced pancreatic β-cell dysfunction, *via* the mediation of PDX1 expression and inhibition of islet inflammation (41). These *in vitro* and animal studies are supported by the observation that DHA-rich fish oil reduced fasting insulin in humans with hyperinsulinemia and insulin resistance (42). These results are supported by the observation that decreased formation of DHA-derived proresolution mediators protectins (including PDX) is associated with insulin resistance because of impaired resolution especially in high fat diet fed experimental animals (43). In a extension of this study, it was reported that PDX has glucoregulatory role that is distinct from its anti-inflammatory action. PDX was found to selectively stimulate the release of IL-6 from skeletal muscle that triggered a myokine-liver signaling axis that blunted hepatic glucose production *via* signal transducer and activator of transcription (STAT3) which suppressed gluconeogenic program (44). In addition, PDX also activated, in an IL-6-dependent manner, AMP-activated protein kinase (AMPK). In *db/db* mice PDX enhanced muscle IL-6 levels that substantially improved their insulin sensitivity (44). These results (44) and the current study data suggest that IL-6 may increase and reduce insulin resistance (45–47) depending on the site of its production. When IL-6 is produced by the skeletal muscle it increases insulin sensitivity whereas its (IL-6) release from adipocytes and macrophages leads to an increase in insulin resistance. How exactly this diametrically opposite actions are produced by IL-6 is not clear. In addition, this dual action of IL-6 may depend not only on its site (tissue) of production but also on the amount released and duration of its release. Furthermore, it was reported that the ability of PDX to trigger release of IL-6 is not dependent on the DHA backbone or on the number, position, and stereochemistry of the hydroxyl groups alone and is not due to the E,Z,E organization of the conjugated triene within the PDX molecule. This led to the suggestion that the ability of PDX to induce the release of skeletal muscle IL-6 is its unique property characterized by its distinct selective skeletal muscle IL-6 secretagogue action (44).

**Figure 5:**
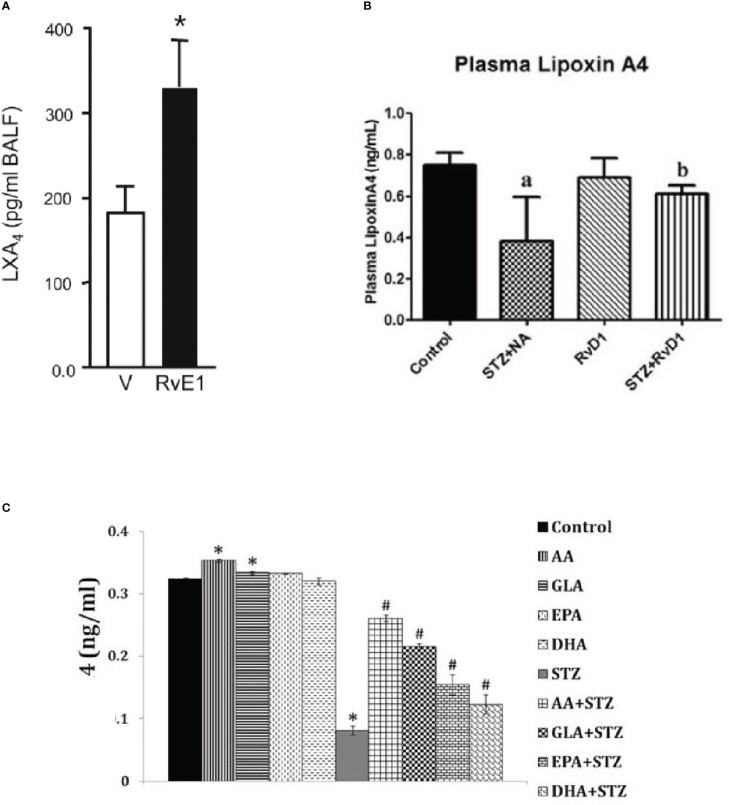
**(A)** Effect of resolvinE1 (derived from EPA) on lxa4 formation in bronchial alveolar fluid f animals with allergic airway inflammation that received 50/100 ng/day of resolvin E1 (this data is takn from reference 50. *P<0.05 compared to vehicle control. **(B)** Effect of resolvin D1 (derived from DHA) give intraperitoneally (60 ng/animal) to STZ induced type 2 DM animals on plasma LXA levels. ^a^P ^<^0.001 compared to untreated control; ^b^P<0.01 compared to STZ+NA (streptozotocin + nicotinamide) treated group. This data is taken from reference 37. **(C)** Effect of various unsaturated fatty acids on the generation of LXA4 inhibited by streptozotocin-treated RIN (rat insulinoma) cells *in vitro*. It is evident that STZ suppressed LXA4 generation by RIN cells that is restored to near normal by AA, while other fatty acids especially EPA and DHA also induced LXA4 generation to a significant degree compared to STZ treated cells but are much less potent compared to AA. RIN cells were treated with STZ (21 mM) and fatty acid concentration used was 10 μg/ml. *P < 0.05 compared to control; ^#^P < 0.05 compared to STZ.

Studies performed in transgenic fat-1 mice that carry a *C. Elegans* gene, fat-1, encoding an n-3 fatty acid desaturase catalysing the conversion of n-6 to n-3 PUFAs (48) (these mice have low concentrations of AA and high amounts of EPA and DHA in their plasma and tissues) revealed that these animals do not develop T1DM when challenged with STZ (49). These animals showed significantly reduced concentrations of pro-inflammatory PGE2 and 12-HETE derived from AA and elevated amounts of anti-inflammatory LXA4 (derived from AA) and 18-HEPE, the precursor of resolvin E1, with a concomitant decrease in IL-6 and TNF-α. These results are in support of our previous studies where we observed that both AA and LXA4 prevent alloxan and STZ-induced T1 and T2 DM (24, 27, 28, 30). Our studies showed that AA is more potent that EPA and DHA in preventing the development of alloxan-induced T1DM (24–26). We and others observed that resolvins enhance the formation of LXA4, a potent anti-inflammatory and anti-diabetic molecule derived from AA. Our *in vitro* studies showed that EPA and DHA also enhance the formation of LXA4 though to a much lesser degree compared to the amount (of LXA4) formed from AA, presumably by displacing AA from the cell membrane lipid pool that, in turn, gets converted to LXA4. In studies that reported the beneficial action of EPA or DHA in DM (40–43) the authors did not measure the plasma or tissue concentrations of LXA4, resolvins or protectins. Hence, it is not certain whether the fatty acids (DHA or EPA or both) by themselves are preventing the development of DM, or it is their anti-inflammatory products resolvins and protectins are responsible for their anti-diabetic action. Based on our previous studies (27–29), we tend to suggest that formation of LXA4/resolvins/protectins from their respective precursors are responsible for the anti-diabetic action of AA, EPA and DHA respectively. Since AA/EPA/DHA enhanced the formation of LXA4 and STZ-treated n-3 transgenic mice showed increased generation of LXA4, it is likely that formation of LXA4 is responsible for the anti-diabetic action of AA/EPA/DHA. The observation that resolvins (derived from EPA and DHA) and EPA and DHA are able to augment LXA4 generation (36, 37) implies that there is a close interaction(s) between n-3 and n-6 fatty acids and their metabolites (see **Figure 5**; 50). Since resolvins that have similar propertied as that of PDX, it is predicted that it (PDX) will be able to enhance LXA4 formation, though this remains to be established.

In general, characteristics of pro-resolving mediators include: limiting or cessation of neutrophil tissue infiltration, regulate the secretion and action of chemokines and cytokines, induce apoptosis of spent neutrophils and their subsequent efferocytosis by macrophages, reprogramming macrophages from M1 (pro-inflammatory) to M2 (anti-inflammatory) type, instruct suppressive immune cells and adaptive immune responses to deal with subsequent encounters, able to induce tissue repair and restore homeostasis (51). In the present study, we did show that PDX is able to suppress IL-6 and TNF-α and enhance IL-10 secretion and able to restore insulin secretion and thus, restore glucose homeostasis. In addition, both LXA4 and PDX have been shown to suppress inappropriate free radical generation (especially ROS), iNOS and COX-2 genes expression (28, 52, 53, see **Figure 4D**). These results suggest that both LXA4 and PDX have inflammation resolution properties, though more definitive studies are warranted particularly regarding PDX.

Since LXA4, resolvins (26–28, 36, 37) and PDX (the present study, and (54) have potent anti-diabetic action (LXA4 > resolvins ≥ protectins) methods need to be developed to exploit them as potential anti-diabetic molecules/drugs. It remains to be seen whether administration of AA/EPA/DHA will suffice to prevent DM and if so, suitable modes of their delivery to humans need to be developed in future.

## Data availability statement

The original contributions presented in the study are included in the article. Further inquiries can be directed to the corresponding author/s. The datasets presented in this study are included in the article.

## Ethics statement

The animal study was reviewed and approved by Ethics committee of GVP Medical College. Written informed consent was obtained from the owners for the participation of their animals in this study.

## Author contributions

UD generated the idea, planned the studies, provided the facilities, supervised the studies, interpreted the data, wrote the manuscript. RP and SP performed the studies, wrote the manuscript. All authors contributed to the article and approved the submitted version.
